# Haplotypic analysis of *cox1* from *Toxocara canis* demonstrates five distinct clades that are not geographically defined

**DOI:** 10.1371/journal.pntd.0011665

**Published:** 2023-10-25

**Authors:** Katy A. Martin, Jeba R. J. Jesudoss Chelladurai, Abrha Bsrat, Cassan Pulaski, Alice C. Y. Lee, Lindsay A. Starkey, Matthew T. Brewer

**Affiliations:** 1 Department of Veterinary Pathology, Iowa State University College of Veterinary Medicine, Ames, Iowa, United States of America; 2 Department of Diagnostic Medicine and Pathobiology, College of Veterinary Medicine, Kansas State University, Manhattan, Kansas, United States of America; 3 Mekelle University College of Veterinary Medicine, Tigray, Ethiopia; 4 Department of Infectious Diseases, College of Veterinary Medicine, University of Georgia, Athens, Georgia, United States of America; 5 Department of Comparative, Diagnostic, and Population Medicine, College of Veterinary Medicine, University of Florida, Gainesville, Florida, United States of America; 6 Department of Pathobiology, College of Veterinary Medicine, Auburn University, Auburn, Alabama, United States of America; Washington University in St Louis School of Medicine, UNITED STATES

## Abstract

**Background:**

*Toxocara canis* is a cosmopolitan parasite of dogs that is transmitted transplacentally to puppies resulting in widespread shedding of eggs in the environment. However, it is not clear if there are dominant parasite genotypes that are more common, pathogenic, or likely to be zoonotic.

**Methods/principle findings:**

Sequences of mitochondrial *cox1* gene from adult worms were used to compare parasites from the United States with submitted sequences from parasites isolated from dogs in different countries. Our analysis revealed at least 55 haplotypes. While we expected the North American worms to form a distinct cluster, we found haplotypes of *T*. *canis* reported elsewhere existing in this population. Interestingly, combining the sequence data from our study with the available GenBank data, analysis of *cox1* sequences results in five distinct clades that are not geographically defined.

**Conclusions:**

The five clades of *T*. *canis* revealed in this study potentially have unique life histories, traits, or host preferences. Additional investigation is needed to see if these distinct clades represent cryptic species with clinically useful attributes or genotypes with taxonomic value. Evaluation of common mitochondrial genes may reveal distinct populations of zoonotic *T*. *canis*.

## Author summary

*Toxocara canis* is a cosmopolitan ascarid nematode of dogs. In humans, following ingestion of eggs, larvae of this parasite cause visceral larva migrans and ocular toxocariasis. *Toxocara canis* continues to persist in the United States, with a high level of seropositivity observed in people. However, relatively little data is available to describe 1) genetic isolates of the parasite that have been transmitted from dogs to humans or 2) geographically isolated clusters of *T*. *canis* that may form taxonomic groups with unique properties. In this study, we collected adult *T*. *canis* from dogs in four different states and applied phylogenetic and haplotypic analyses to compare parasite *cox1* sequences with those reported globally. Our hypothesis was that we would discover new haplotypes that were unique to the United States. However, our data demonstrated very little geographic clustering of isolates. Instead, maximum likelihood trees and median joining networks supported the conclusion that nearly all haplotypes reported thus far worldwide fell within 5 distinct clades. Analysis of additional genes and parasite traits could reveal distinct clades of *T*. *canis* with specific clinical significance such as those predisposed to causing disease in humans.

## Introduction

*Toxocara canis* is a common nematode parasite of canines throughout the world. Adult worms live in the small intestine of dogs and lay eggs that are passed in feces. Upon ingestion of the larvated egg by susceptible dogs, larvae hatch and undergo a blood-liver-lung migration prior to being adult nematodes. However, a proportion of larvae migrate into the somatic tissues where they become arrested as hypobiotic larvae. These tissue-dwelling larvae are reactivated during the third trimester of pregnancy, crossing the placenta to infect puppies. This mechanism of ubiquitous infection has led to a widespread need for anthelmintic treatment in dogs. Toxocariasis is designated as one of five Neglected Parasitic Infections by the CDC based the number of infections, severity of illness, and the ability to prevent and/or treat infections [1]. In humans, migrating larvae cause visceral and ocular larval migrans [2]. A 2018 study estimated that 5% of the U.S. population is seropositive for *Toxocara* antibodies [3].

While *T*. *canis* is a common parasite of domestic dogs worldwide, there is relatively little data available on the population genetic structure of this organism. Understanding the phylogenetic relationship of pathogens can be important for clinical purposes as it may provide information about drug resistance, virulence, or other relevant parameters [4,5]. In similar nematodes such as *Ascaris suum*, molecular epidemiological studies have utilized nuclear and mitochondrial sequences to investigate population structuring and haplotypic variation [6–9]. For example, studies of *A*. *suum* have revealed geographic clustering of sequence relationships and potential zoonotic transmission events [6]. However, few or no studies have been undertaken to describe such population traits in *T*. *canis* in the United States.

A recent study used the cytochrome c oxidase subunit 1 mitochondrial gene (*cox*1) to examine the relationship between *T*. *canis*, *T*. *cati*, *T*. *malaysiensis*, and *Toxascaris leonina* from 8 countries: Brazil, China, Denmark. Germany, Japan, Malaysia, Portugal, and Russia [10]. Fava et al found that sequences grouped based on host species (canids and felids) but did not exhibit any geographic relationship. The authors suggest gene flow among populations due to global movement of animals as the reason for the lack of geographic clustering of specimens [10].

In the present study, we aimed to understand genetic and haplotypic diversity of *T*. *canis* adults isolated from dogs in the United States using the partial mitochondrial barcoding *cox1* gene and compare it with those reported globally. We hypothesized that the geographical isolation of North America would drive haplotypic differentiation and structuring in *T*. *canis* populations in the U.S. and those reported from other global regions.

## Materials and methods

### Ethics statement

The research was approved by the Iowa State University Institutional biosafety committee #21289.

### Parasites

*Toxocara canis* adult specimens were collected by licensed veterinarians from dogs in Alabama (28 samples), Florida (14 samples), Iowa (8 samples), and Louisiana (19 samples) from 2017–2019. Species identity of the parasites were established using morphological features as identified by practicing veterinary parasitologists (the authors) and submitted to the Iowa State University College of Veterinary Medicine. The samples were collected opportunistically; any morphologically bona fide *Toxocara* from a dog was eligible for inclusion in the study. When possible, efforts were made to collect only 1 worm per canine host in order to search for greater genetic diversity. Parasites were identified using traditional keys [11] and stored in 70% ethanol until the time of DNA extraction. All protocols were approved by the Institutional Bio-safety Committee at Iowa State University 15-I-0027-A/H.

Sterile surgical blades were used to remove a 5 mm portion of tissue from the anterior end of each worm. DNA was extracted using the Qiagen DNeasy Blood and Tissue kit (Valencia, CA) according to manufacturer’s instructions. Genomic DNA was eluted in 100 μL of water and stored at -20°C.

### Amplification of *cox1*

An approximately 425-bp fragment of the mitochondrial cytochrome oxidase 1 (*cox1*) gene was amplified using the primers ToxCoIF (5′-GATTTTACCTGCTTTTGGTATTATTAG-3′) and ToxCoIR (5′-CCAAAGACAGCACCCAAACT-3′) [10]. PCR was carried out in a 20 μL volume with 2 μl DNA template, 1x PCR buffer, 2 mM MgCl_2_, 0.5 μM of each dNTP, 0.3 μmol of each primer, and 1 U of Taq polymerase (GoTaq Flexi, Promega, Madison WI). PCR conditions consisted of initial denaturation at 95°C for 3 min, followed by 35 cycles: 30s denaturation (95°C), 40s annealing (60°C), 1 min elongation (72°), and 5 min step of final elongation (72°C). Agarose gel electrophoresis was performed to confirm amplification and the PCR product was sequenced on an Applied Biosystems 3730xl DNA Analyzer. DNA sequences were assembled using CAP3 [12] (available at: http://doua.prabi.fr/software/cap3). Sequences are available in the GenBank nucleotide database (accession numbers OP131517 –OP131578).

### Phylogenetic and haplotypic analysis

A total of 63 new sequences from this study were acceptable for phylogenetic and haplotypic analysis. Additionally, sequences from Fava et al (2020)[10] and others available on GenBank were included. Blast searching, searching of FASTA files from *Toxocara* transcriptomes, and multiple sequence alignment was used to identify potential sequences. All sequences available on GenBank consisting of the 327 paired nucleotides were included in the analysis. The final dataset consisted of 135 sequences with 63 isolates from the United States, 1 from Australia, 12 from Brazil, 6 from China, 11 from Denmark, 5 from Germany, 24 from Iran, 1 each from Japan, Netherlands, Poland, Portugal and 9 from Russia. Sequences were trimmed to 327 base pairs and alignment was performed using ClustalW on MegaX [13].

Mega-X [13] was used to infer evolutionary history and generate maximum likelihood trees using the General Time Reversible model with a gamma distribution [14] and 1000 bootstrap replications. *Toxocara cati* (Accession number: MT942618.1) was used as an outgroup. Trees were constructed both with and without sequences from previous studies [10] and other *T*. *canis cox1* sequences available on GenBank.

Haplotype analysis was performed using DNASp v6 software [15]. A median joining haplotype network [16] was constructed in Population Analysis with Reticulate Trees (PopArt 1.7) [17] (available at: http://popart.otago.ac.nz). Additionally, a haplotype consensus maximum likelihood tree was constructed in MegaX.

## Results

### Identification of *T*. *canis* and sequence analysis

All PCR reactions generated an amplicon of the expected size except 4 (1 Florida, 3 Iowa) which were excluded from the analysis. NCBI BLAST analysis was used to confirm sequences were consistent with *T*. *canis*. One specimen (Iowa) blasted as *Baylisascaris* and was removed from the remainder of the analysis. Additionally, one specimen (Alabama) was removed from the analysis due to poor sequence quality.

### Phylogenetic analysis of *cox1* sequences

**Fig 1:**
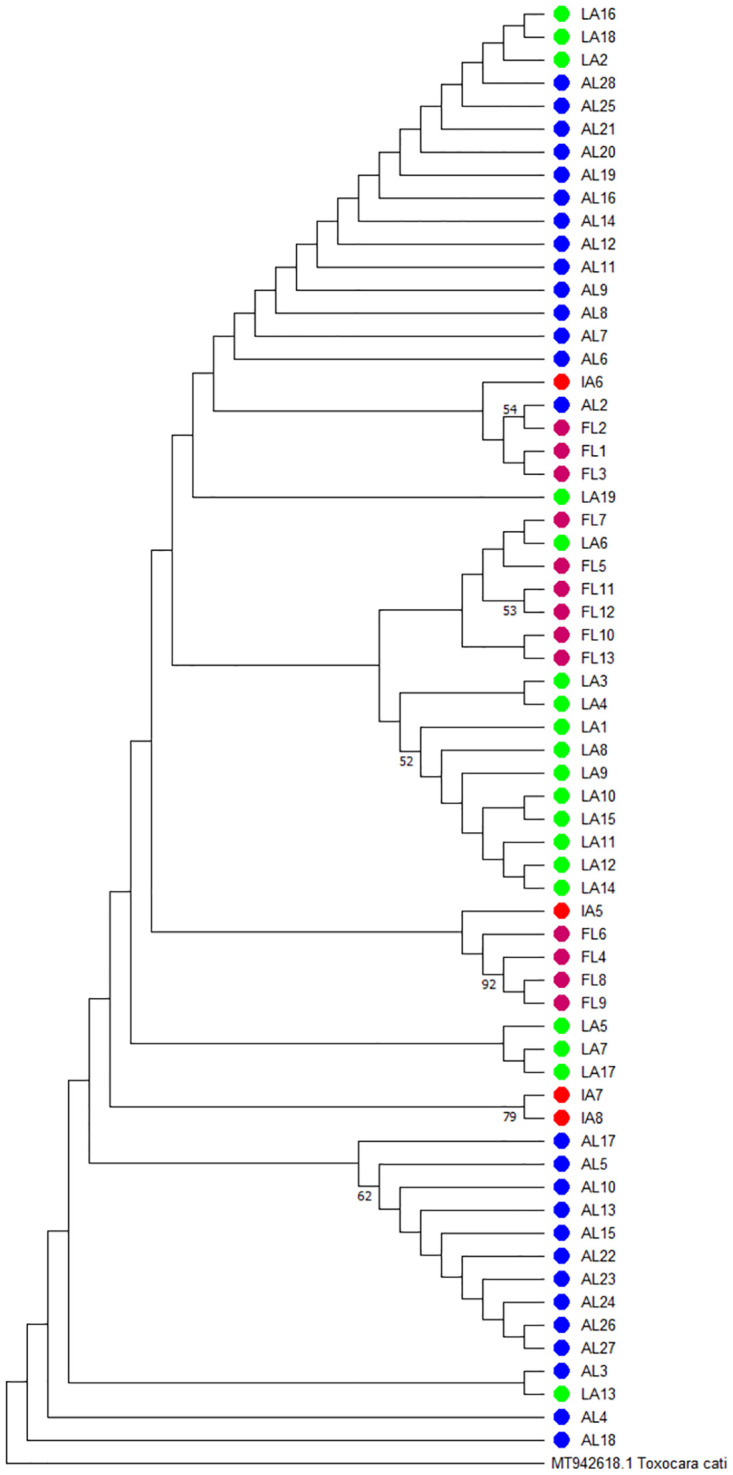
Maximum likelihood using *T*. *canis* samples collected in this study from the United States. Branches with greater than 50% bootstrap support are indicated with the corresponding bootstrap value.

**Fig 2:**
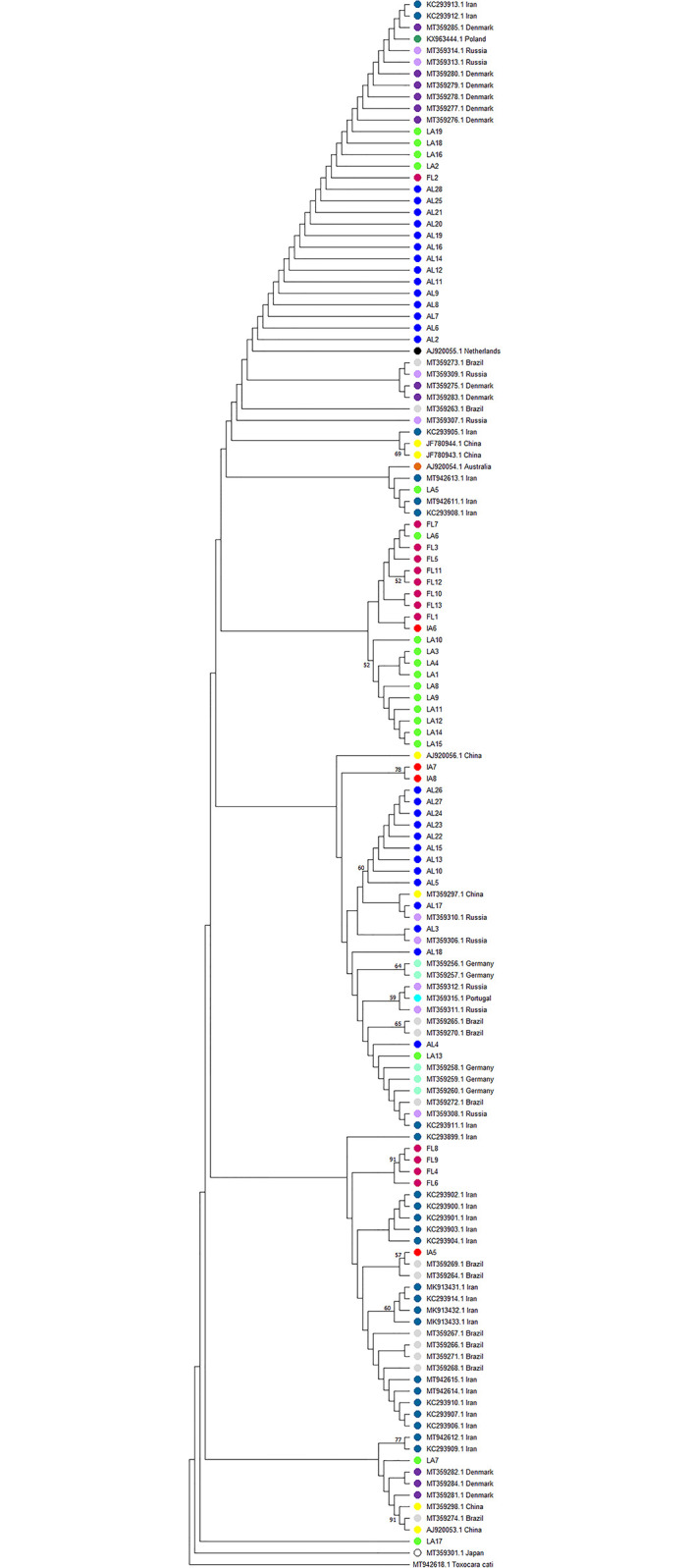
Maximum likelihood using both sequences from this study and global sequences available on Genbank. Branches with greater than 50% bootstrap support are indicated with the corresponding bootstrap value.

A maximum likelihood tree constructed using the sequences collected in this study as well as a tree constructed using additional sequences available in GenBank, including those from Fava et al [10] (GenBank accession numbers: MT359256 –MT359318), are shown in Figs 1 and 2, respectively. When analyzed on their own, the sequences from our study appeared to form loose clusters primarily made up of samples from Alabama, Florida, and Louisiana while samples from Iowa shared common characteristics from several other states (Fig 1). On the other hand, unexpectedly, when a global analysis was performed on all sequences in GenBank, samples from different continents clustered together (Fig 2). A maximum likelihood analysis using amino acid sequences yielded similar results (S1 and S2 Figs).

### Haplotype analysis of *cox1* sequences

**Fig 3:**
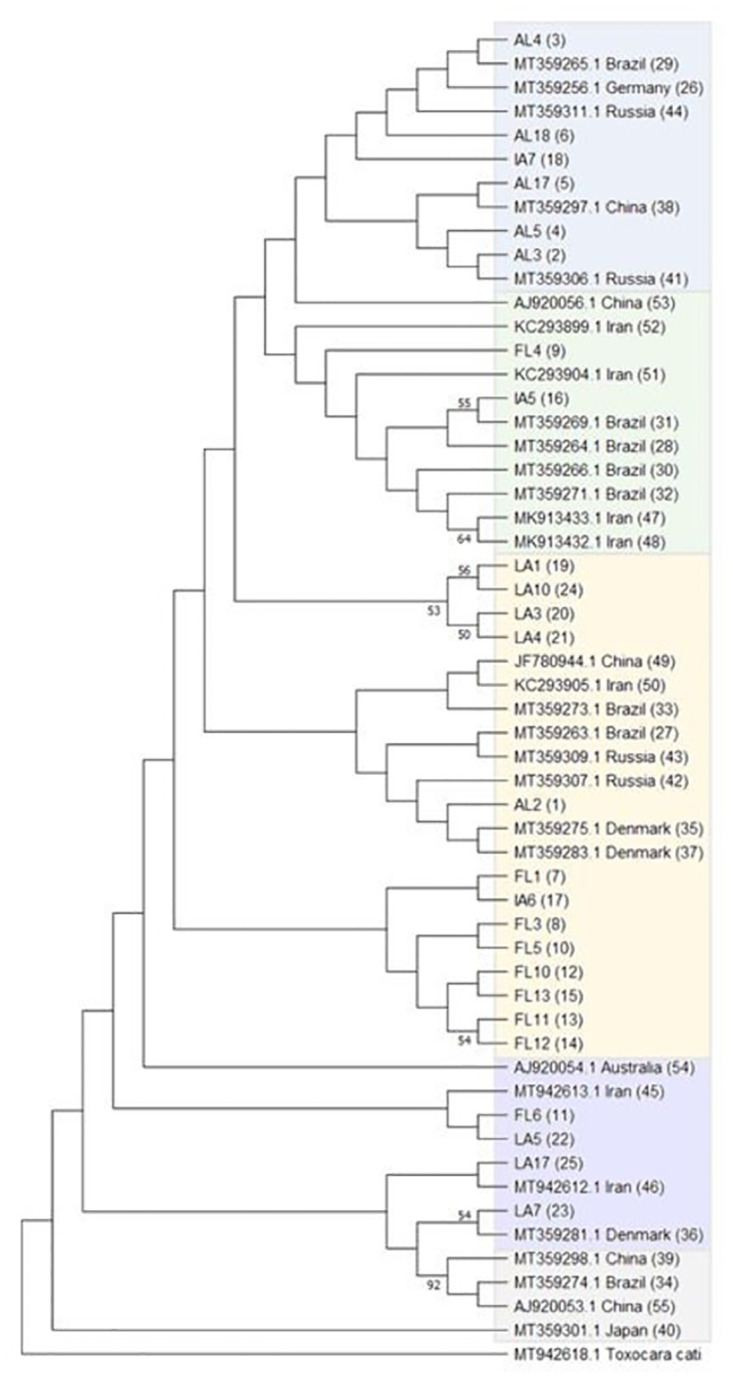
Haplotype consensus maximum likelihood tree from all available *T*. *canis cox1* sequences in GenBank; haplotypes are given in parentheses. Haplotypic clusters are colored to match the median joining network in Fig 4. Branches with greater than 50% bootstrap support are indicated with the corresponding bootstrap value.

**Fig 4:**
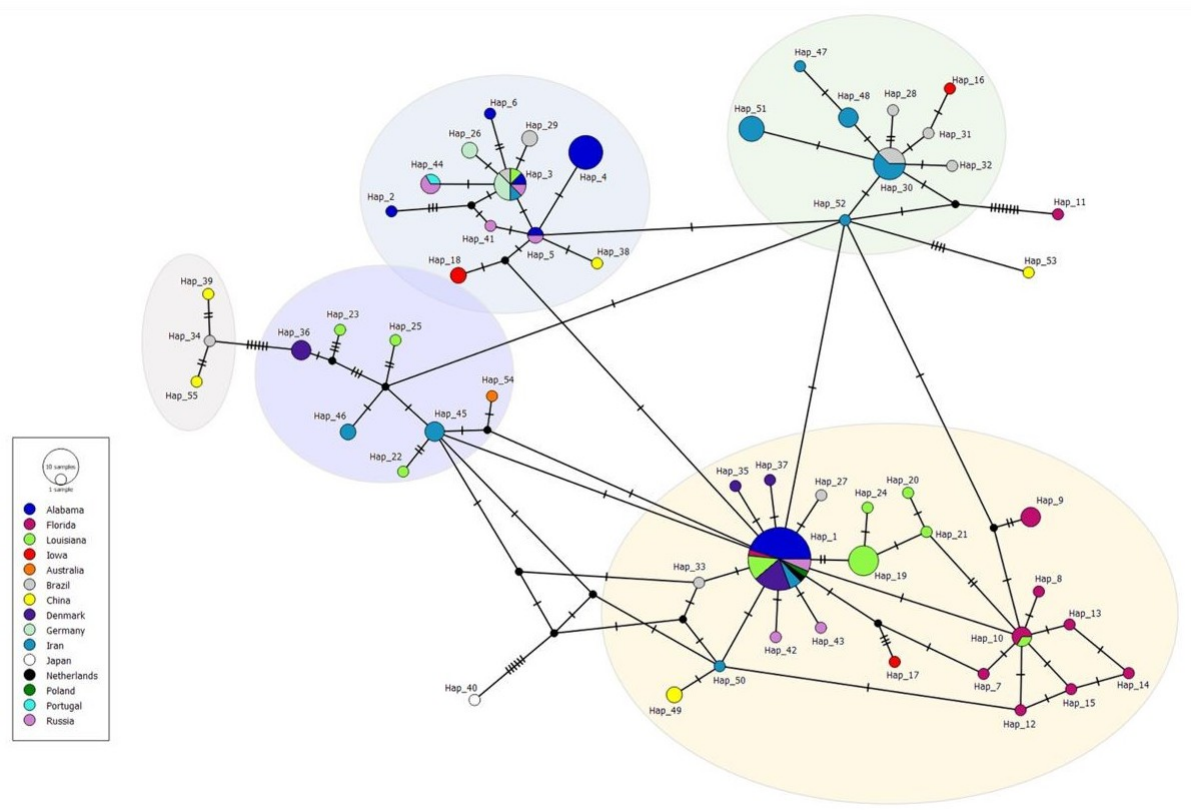
Median joining network of *T*. *canis* haplotypes found worldwide. Haplotypic clusters are colored to match the maximum likelihood tree in Fig 3. Colored circles represent individual haplotypes. Hash marks indicate the number of nucleotide differences between different haplotypes.

Haplotype analysis of 135 sequences revealed 55 haplotypes with a haplotypic diversity of 0.932, nucleotide diversity of 0.01227, and 54 segregating variant sites (Figs 3 and 4). We designated the predominant haplotype as haplotype 1. Table 1 provides descriptions of the haplotypes including haplotype frequency, sequences included in each haplotype, and the country of origin for each sequence. Haplotypes demonstrated geographic grouping, with the majority (50 out of 55) of haplotypes consisting of sequences from one location. Haplotype 1 contains sequences from the U.S. (Alabama, Florida, Louisiana), Denmark, Russia, Iran, Poland and Netherlands. Haplotypes 3 and 5 were the only other shared haplotype between the U.S. and Europe. Haplotypes 4, 6–25 were novel and unique to the U.S. Haplotype 4 consisted entirely of sequences from Alabama; haplotype 9 of sequences entirely from Florida and haplotype 19 of sequences from Louisiana (Table 1 and Fig 4). A maximum likelihood analysis using amino acid sequences yielded similar results (S3).

**Table 1:** Global haplotype analysis for *cox1* of *T*. *canis*.

Haplotype	Haplotype frequency	Sequence ID	Country of Origin
1	0.23	AL2, AL6, AL7, AL8, AL9, AL11, AL12, AL14, AL16, AL19, AL20, AL21, AL25, AL28, FL2, LA2, LA16, LA18, LA19	USA
		MT359276.1, MT359277.1, MT359278.1, MT359279.1, MT359280.1, MT359285.1	Denmark
		MT359313.1, MT359314.1	Russia
		KC293913.1, KC293912.1	Iran
		KX963444.1	Poland
		AJ920055.1	Netherlands
2	0.01	AL3	USA
3	0.06	AL4	USA
		LA13	USA
		MT359258.1, MT359259.1, MT359260.1	Germany
		MT359272.1	Brazil
		MT359308.1	Russia
		KC293911.1	Iran
4	0.07	AL5, AL10, AL13, AL15, AL22, AL23, AL24, AL26, AL27	USA
5	0.01	AL17	USA
		MT359310.1	Russia
6	0.01	AL18	USA
7	0.01	FL1	USA
8	0.01	FL3	USA
9	0.02	FL4, FL8, FL9	USA
10	0.02	FL5, FL7, LA6	USA
11	0.01	FL6	USA
12	0.01	FL10	USA
13	0.01	FL11	USA
14	0.01	FL12	USA
15	0.01	FL13	USA
16	0.01	IA5	USA
17	0.01	IA6	USA
18	0.01	IA7, IA8	USA
19	0.05	LA1, LA8, LA9, LA11, LA12, LA14, LA15	USA
20	0.01	LA3	USA
21	0.01	LA4	USA
22	0.01	LA5	USA
23	0.01	LA7	USA
24	0.01	LA10	USA
25	0.01	LA17	USA
26	0.01	MT359256.1, MT359257.1	Germany
27	0.01	MT359263.1	Brazil
28	0.01	MT359264.1	Brazil
29	0.01	MT359265.1, MT359270.1	Brazil
30	0.06	MT359266.1, MT359267.1, MT359268.1	Brazil
		MT942615.1, MT942614.1, KC293910.1, KC293907.1, KC293906.1	Iran
31	0.01	MT359269.1	Brazil
32	0.01	MT359271.1	Brazil
33	0.01	MT359273.1	Brazil
34	0.01	MT359274.1	Brazil
35	0.01	MT359275.1	Denmark
36	0.02	MT359281.1, MT359282.1, MT359284.1	Denmark
		MT359282.1	Denmark
		MT359284.1	Denmark
37	0.01	MT359283.1	Denmark
38	0.01	MT359297.1	China
39	0.01	MT359298.1	China
40	0.01	MT359301.1	Japan
41	0.01	MT359306.1	Russia
42	0.01	MT359307.1	Russia
43	0.01	MT359309.1	Russia
44	0.02	MT359311.1, MT359312.1	Russia
		MT359315.1	Portugal
45	0.02	MT942613.1, MT942611.1, KC293908.1	Iran
46	0.01	MT942612.1, KC293909.1	Iran
47	0.01	MK913433.1	Iran
48	0.02	MK913432.1, MK913431.1, KC293914.1	Iran
49	0.01	JF780944.1, JF780943.1	China
50	0.01	KC293905.1	Iran
51	0.04	KC293904.1, KC293903.1, KC293901.1, KC293902.1, KC293900.1	Iran
52	0.01	KC293899.1	Iran
53	0.01	AJ920056.1	China
54	0.01	AJ920054.1	Australia
55	0.01	AJ920053.1	China

Genetic differentiation between U.S. sequences and non-U.S. global sequences was low with an Fst of 0.08309. Fst between U.S. sequences and European, Asian, and South American sequences were 0.0569, 0.12218, and 0.12728 respectively. Distance between US sequences and Australian sequences were could not be calculated due to the paucity of data (1 sequence) from Australia.

A haplotype consensus tree was constructed using MegaX and is shown in Fig 3. This analysis also supported the conclusion that haplotypic clustering was not geographically driven and resulted in the identification of ~5 haplotypic clades (Fig 3). A maximum likelihood analysis using amino acid sequences yield similar results (S3 Fig). Interestingly, when evaluating the median joining network, we also observed 5 main clusters (Fig 4). We found that the majority of haplotypes (51/55) group in the same clusters in the median joining network as when analyzed in the maximum likelihood tree, but these relationships were not geographically defined. Three haplotypes: 11, 40, and 53 fell outside of the 5 clusters in the median joining network as they are separated by 4 or more nucleotide changes (Fig 4).

## Discussion

*Toxocara canis* is an important parasite due to its impact on both animal and human health. In this study, we investigated the haplotypes of *T*. *canis* in the United States and compared them to the haplotypic sequences available for this parasite worldwide. The evolutionary/genetic relationship of parasites can serve as a framework for understanding trends in anthelmintic resistance, virulence, host predilection, and zoonotic potential. For example, phylogenetic and haplotypic analysis was used to infer the zoonotic potential of the zoonotic parasite *Strongyloides stercoralis*. Genes, including *Cox1*, revealed two lineages of *S*. *stercoralis*, whereby lineage A was zoonotic but lineage B was thought to be an ancestral genotype which is not adapted to humans [18]. Similarly, phylogenetic approaches utilized for *Plasmodium* parasites have provided insights regarding the significant variation in clinical disease across host species [19]. These examples provide support for the importance of investigating the phylogenetic relationships of parasites. Our study is the first depositing *cox*1 sequences and haplotypes from the United States.

The present study provides a basic understanding of *T*. *canis* haplotypes in the United States, but much could be revealed through more extensive sampling. Genetic studies of many veterinary parasites are conducted as specimens are obtained opportunistically; collecting adult worms is logistically challenging for a variety of reasons. Also, as Fava [10] hypothesized, movement of animals and therefore their parasites could be influencing the phylogeographic results measured by DNA sequencing data. While the approximate location of the animal at the time of specimen collection was recorded and utilized for the present analysis, no information regarding previous travels of the animals was collected. Similarly, our state-level data is not amenable to a precise analysis. Still, this study demonstrated that even opportunistic sampling reveals interesting traits about the haplotypes found in the United States and globally.

The *cox1* gene has been described as a more ideal target for phylogeographic analysis due to a relatively high mutation rate, intraspecific variability, and maternal inheritance [20]. In this study, we expected to find new *cox1* haplotypes since there is a paucity of data available for North America. However, this was not the case: our results suggest that haplotypes reported abroad were also present in the United States. Moreover, analysis of median joining networks and maximum likelihood analysis of consensus haplotypes both sorted parasites into five main clades. Interestingly, 51/55 haplotypes fell in the same predicted clades in both analyses. Thus, our results suggest that populations of *T*. *canis* with specific characteristics may be distinguishable on a global scale. Whether or not these populations represent cryptic species is a matter of speculation at this time, we refrain from naming them with any significant nomenclature. While our finding of specific clades of *T*. *canis* is enticing, further clinical or life cycle traits can be correlated with our sequence-based clades. Indeed, such labels should only be pursued if they are useful for scientists and clinicians [21].

While our finding of specific clades of *T*. *canis* is enticing, one limitation of the study was the lower bootstrap support in the maximum likelihood trees which indicates a decreased amount of variation in this gene from the population under study. A different mitochondrial gene, such as *nad4*, or analysis of multiple genes may provide a higher level of detail to allow for the elucidation of phylogeographic relationships that may exist [22]. Indeed, a concatenation of data from multiple genes can be used to clarify the picture of nematode phylogeny [23]

In the present study, we investigated populations of *T*. *canis* from the United States. Sequencing of *cox1* sequences enabled us to deposit new GenBank sequences from specimens in the United States and make comparison with those available worldwide. Using phylogenetic analysis, we found that *cox1* sequences were moderately supportive of clustering, whereby 5 clades were present throughout the world. However, this clustering could not be explained solely by geography. Additional studies are needed to determine if the various haplotypes have unique life history or clinical traits worthy of attention by clinicians. In summary, this study contributes a broader understanding of *T*. *canis* haplotypes and provides a basis for future genetic studies of the parasite that identify zoonotic haplotypes found in human infection.

## Supporting information

S1 Fig Maximum likelihood tree constructed using amino acid sequences of *T*. *canis* collected in this study. (DOCX)

S2 Fig Maximum likelihood tree constructed using amino acid sequences of *T*. *canis* collected in this study and global sequences available on Genbank. (DOCX)

S3 Fig Maximum laikelihood tree constructed using amino acid sequences of consensus haplotypes. (DOCX)
