# Supplementary material for: Haplotypic analysis of cox1 from Toxocara canis demonstrates five distinct clades that are not geographically defined
Source: PLoS Negl Trop Dis. 2023 Oct 25;17(10):e0011665. doi: 10.1371/journal.pntd.0011665 (PMC10599572; doi:10.1371/journal.pntd.0011665)
Supplement: S3 Fig — (DOCX) [file pntd.0011665.s003.docx]

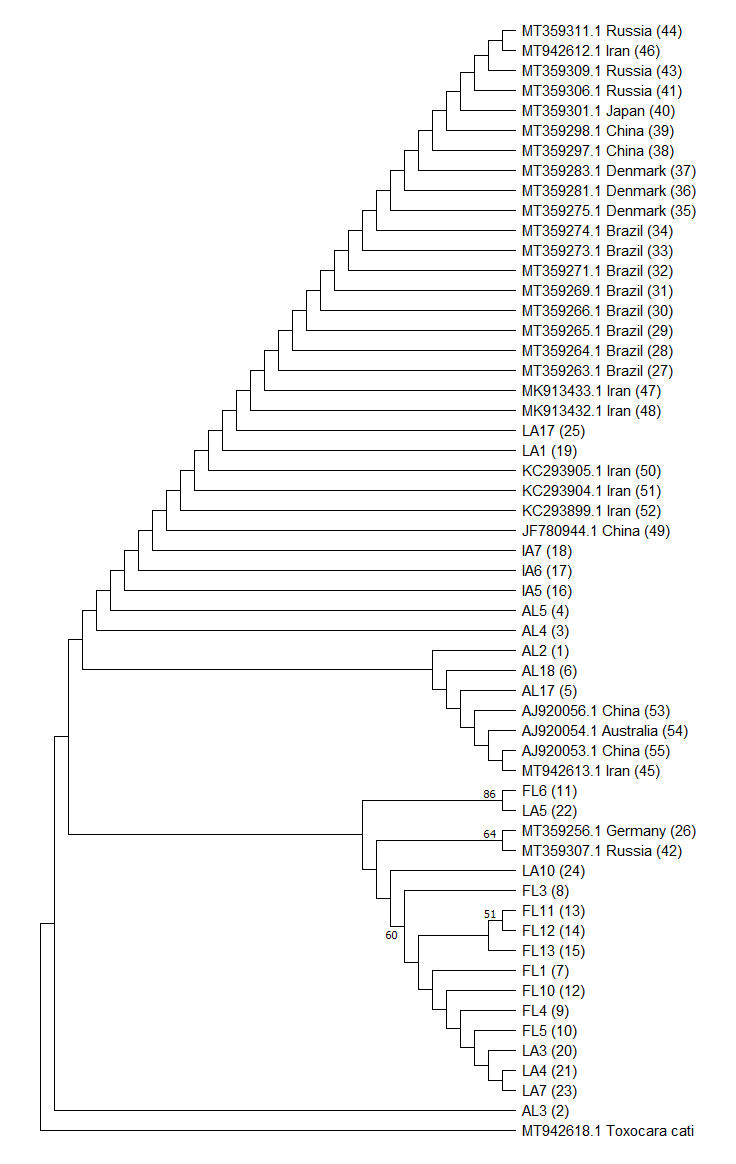


Supplemental figure 3. Maximum likelihood tree constructed using amino acid sequences of consensus haplotypes.
